# Immunorecognition and Neutralization of *Crotalus durissus cumanensis* Venom by a Commercial Antivenom Produced in Colombia

**DOI:** 10.3390/toxins14040235

**Published:** 2022-03-25

**Authors:** Augusto Acosta-Peña, Vitelbina Núñez, Jaime Andres Pereañez, Paola Rey-Suárez

**Affiliations:** 1 Programa de Biología, Pontificia Universidad Javeriana, Bogotá 110231, Colombia; acosta.augusto@javeriana.edu.co; 2 Grupo de Investigación en Toxinología, Alternativas Terapéuticas y Alimentarias, Facultad de Ciencias Farmacéuticas y Alimentarias, Universidad de Antioquia, Medellín 050010, Colombia; vitelbina.nunez@udea.edu.co; 3 Escuela de Microbiología, Universidad de Antioquia, Medellín 050010, Colombia; 4 Centro de Investigación en Recursos Naturales y Sustentabilidad, Universidad Bernardo O’Higgins, Santiago 8320000, Chile

**Keywords:** antivenomics, immune reactivity, Colombia, snakebite, antivenom therapy, *Crotalus durissus cumanensis*

## Abstract

In Colombia, on average 2.9% of the nearly 5600 snakebite events that occur annually involve the rattlesnake *Crotalus durissus cumanensis*. The envenomation by this snake is mainly characterized by neurotoxicity and the main toxin is crotoxin (~64.7% of the total venom). The Instituto Nacional de Salud (INS) produces a polyvalent antivenom aimed at the treatment of bothropic, crotalid, and lachesic envenomations; nonetheless, its immune reactivity profile and neutralizing capacity over biological activities of the *C. d. cumanensis* venom has been poorly evaluated. In this sense, the study aims: (1) to describe an in-depth exploration of its immunoreactivity through second-generation antivenomics and HPLC fraction-specific ELISA immunoprofiles; and (2) to evaluate the neutralization pattern of the rattlesnake venom in vitro and in vivo biological activities. The results obtained showed a variable recognition of crotoxin subunits, in addition to a molecular mass-dependent immunoreactivity pattern in which the disintegrins were not recognized, and snake venom metalloproteinases and L-amino acid oxidases were the most recognized. Additionally, a high neutralization of proteolytic and coagulant activities was observed, but not over the PLA_2_ activity. Further, the median effective dose against *C. d. cumanensis* venom lethality was 962 μL of antivenom per mg of venom. In conclusion, (1) the antivenom recognition over the crotoxin and the disintegrins of the *C. d. cumanensis* should be improved, thus aiming upcoming efforts for the exploration of new techniques and approaches in antivenom production in Colombia, and (2) the neutralization activity of the antivenom seems to follow the molecular mass-dependent recognition pattern, although other explanations should be explored.

## 1. Introduction

Snake venoms, as an evolutionary strategy for predation, are commonly compared with complex cocktails due to their highly diverse structure, composition, and function [1]. Venom has been shown to vary along with snakes’ taxonomical classification, geographical distribution, sex, diet, and ontogenetic state, both at intraspecific and interspecific levels [2,3,4,5,6].

The biochemical heterogeneity of venoms determines a wide range of clinical manifestations that occur when snakes inject it into humans Snakebite envenoming has been categorized by the World Health Organization (WHO) as a Neglected Tropical Disease (NTD) annually affecting nearly 2.7 million people, and causing between 81,000 and 138,000 deaths, along with approximately 400,000 surviving victims suffering associated chronic morbidity, physical disabilities, and psychological sequels [7,8,9,10]. This disease, usually caused by accidental events, perpetuates the cycle of poverty by mostly affecting tropical communities poorly developed, politically marginalized, and whose work activities take place in the field [7,11]. In Colombia, it is estimated that nearly 5600 events occur annually, of which approximately 2.9% involve the Colombian rattlesnake *Crotalus durissus cumanensis* [12].

The venom of the rattlesnake distributed in Colombia, *C. d. cumanensis*, compose of a variety of proteins: crotoxin (CTX, 64.7% of the total venom), disintegrins (DSIs, 13.7%), crotamines (CTA, 5.8%), serine proteinases (SPs, 6.3%), snake venom metalloproteinases (SVMPs, 3.3%), L-amino acid oxidases (LAAOs, 3.2%), cysteine-rich secretory proteins (CRISP, 1.3%), C-type lectins (CTL, 1.2%), and phospholipases A_2_ (PLA_2_, 0.6%) [13]. The synergistic action of these toxins triggers an envenomation with local and systemic actions that develop nephrotoxic, hemostatic, myotoxic, and, predominantly, neurotoxic effects [12]. The neurotoxicity of the *C. d. cumanensis* venom is characterized by flaccid paralysis in the peripheral, facial, ocular, and respiratory musculature [12]. Moreover, the venom median lethal dose (LD_50_) is 1.8 μg/mouse and corresponds to the lowest of Colombia snake venoms described so far [14]. The neurotoxic, myotoxic, and nephrotoxic activities of *C. d. cumanensis* venom are mainly attributed to the crotoxin, a toxin formed by a basic PLA_2_ (CB) and an acidic subunit (Crotapotin) [15,16].

Antivenoms are the only scientifically validated effective treatment for snakebite envenoming and comprise concentrated immunoglobulins commonly raised in horses against a venom -monovalent- or multiple venoms -polyvalent- from a particular geographical area [7,17]. Three antiviperid polyvalent antivenoms are commercialized in Colombia with a high frequency: two are produced within the country, one by the Instituto Nacional de Salud (INS) and the other by Laboratorios Probiol S. A.; and additionally, one that is imported from Mexico (Instituto Bioclón) [18].

Despite being the only specific treatment for snakebite envenoming, antivenom therapy safeness, efficacy, and effectiveness [19] have four major problems: (1) limited reversal of pre-synaptic neurotoxicity (such as the caused by *C. d. cumanensis*); (2) inability to preclude irreversible tissue damage (e. g., necrosis); (3) high frequency of allergic reactions and serum sickness; and the (4) requirement of high doses, high production costs and the instability of the antivenom market makes it non-affordable for the patients and a serious economic load for the governments [7,11,19,20,21,22,23]. Thus, a detailed understanding of the neutralizing and immunoreactivity profiles of the antivenoms needs to be achieved to help solve these problems [24].

In this sense, over the years, various techniques have been developed for the evaluation of antivenoms’ ability to recognize and neutralize venom proteins [25]. The inclusion of proteomics has been of relevant significance as it has provided quantitative assays capable of identifying the presence and relative abundance of toxins in whole venoms (“venomics”), and in fractions recognized and non-recognized by the antivenoms, which is called “antivenomics” [26,27]. The second and third generations of antivenomics, based on immunoaffinity chromatography, provide better resolved proteomic profiles in comparison with the immunodepletion-based first-generation [28]. In coupling to immunological approaches, in vitro and in vivo assessments serve as a powerful tool to evaluate antivenom efficacy in neutralizing the biological activities of venoms [25].

In regard to the INS polyvalent antivenom, (1) pre-clinical and/or clinical evaluations have been done on the neutralization of the activities of *Bothrops*, *Porthidium*, *Lachesis*, and *Bothriechis* species venoms [29,30,31,32,33,34]; and (2) the stability of its immunoreactivity has been tested against *C. d. cumanensis* the whole venom over a time and temperature gradient [35]. Additionally, the *C. d. cumanensis* venom from Colombia has also been tested with Antivipmyn TRI -an antivenom produced in Mexico- using first-generation antivenomics [13]. Nonetheless, specific information regarding the Colombian antivenoms’ immunoreactivity over *C. d. cumanensis* venom proteins and neutralization over biological activities is still scarce.

Therefore, to enhance the safeness and effectiveness of crotalid snakebite treatment in Colombia it is still needed to produce more precise information that allows the foundation of a base for the development of future strategies for the improvement of antivenoms [17,28]. In this sense, the aim of this study is to describe the immunorecognition pattern and to evaluate the neutralizing capacity of biological activities of the *C. d. cumanensis* venom by one commercial antivenom produced in Colombia.

## 2. Results

### 2.1. Immunoreactivity Assessment

**Figure 1:**
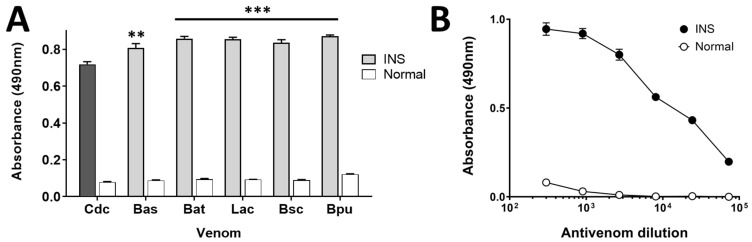
Immunorecognition of the INS antivenom against the venoms of different Colombian vipers and *C. d. cumanensis* venom. In (**A**), ELISA of the whole venom of Cdc: *Crotalus durissus cumanensis;* Bas: *Bothrops asper*; Bat: *B. atrox*; Lac: *Lachesis acrochorda*; Bsc: *Bothriechis schlegelii;* and Bpu: *B. punctatus*. INS and Normal refer to the treatment with the antivenom and with the immunoglobulins of pre-immunized horses, respectively. ** (*p* < 0.05) and *** (*p* < 0.001) represents statistical differences respect to Cdc (darker column). In (**B**), ELISA of the whole *C. d. cumanensis* venom against the INS polyvalent antivenom. (*n* = 3). Each point represents the mean ± SD.

The INS antivenom showed reactivity over *B. asper*, *B. atrox*, *L. acrochorda*, *B. schlegelii*, *B. punctatus*, and *C. d. cumanensis* venoms. However, against the latter showing the lowest levels of recognition (Figure 1A). And, even so, antibody titers against *C. d. cumanensis* venom were observed up to the lowest tested concentration (Figure 1B, *p* < 0.05).

**Figure 2:**
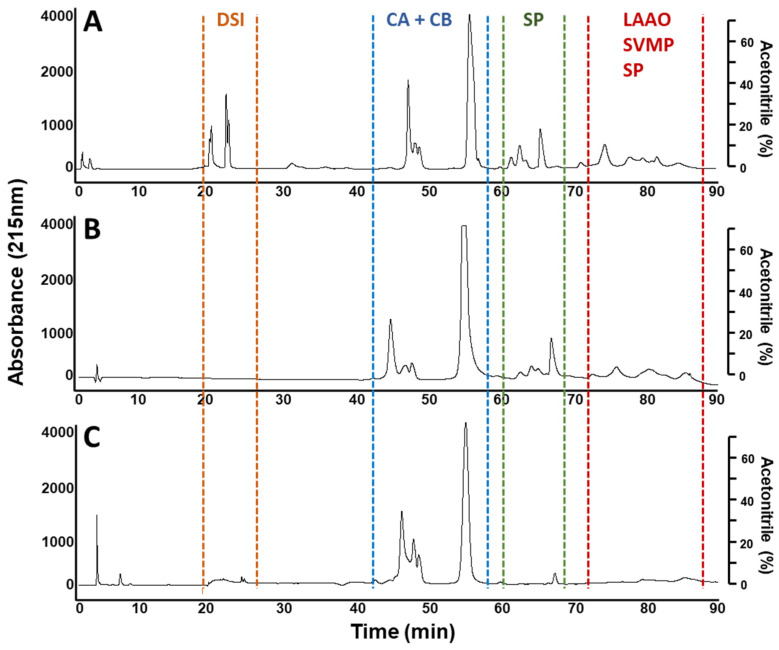
Colombian INS antivenom immunorecognition of *C. d. cumanensis* venom proteins by second-generation antivenomics. The RP-HPLC profiles of the whole venom and the fractions of snake venom proteins retained and not retained by antivenom IgGs immobilized in the affinity matrix. (**A**), whole venom; (**B**), retained fraction; (**C**), not-retained fraction. According to the previous venomic characterization of *C. d. cumanensis* by Quintana-Castillo, et al. (13), four main regions are distinguished in the chromatograms, for which main protein families have been identified. DSI: Disintegrin region (yellow); CA and CB: Crotoxin A and Crotoxin B region (blue); SP: Serine Proteinase region (green); and SVMP, CTL and LAAO: Snake Venom Metalloproteinase; C-type lectin and L- amino acid oxidase region (red).

**Figure 3:**
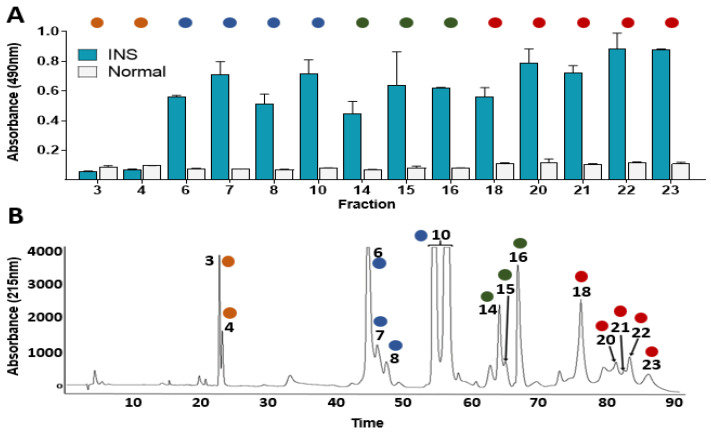
ELISA-based immunoprofile of INS antivenom against RP-HPLC fractions of *C. d. cumanensis* venom. In (**A**), ELISA, immunoreactivity of the INS antivenom over the venom RP-HPLC fractions. In (**B**), RP-HPLC profile of the whole venom from which the fractions were collected, the peaks are numbered. Pre-immunized horse serum was used as a negative control (Normal). The code of colors is the same as in Figure 2.

The whole venom chromatography regions obtained in this study were associated with the proteins identified previously [13]. In this sense, the second-generation antivenomics and the ELISA based immunoprofile results showed a recognition ability pattern of the INS polyvalent antivenom lying towards the *C. d. cumanensis* venom proteins eluted in the last regions of the chromatogram (L-amino acid oxidase, LAAO; Serine Proteinase, SP; C-type lectin, CTL; Snake Venom Metalloproteinase, SVMP) whereas the low retention time proteins were poorly recognized (Figure 2 and Figure 3). Particularly, the HPLC peaks corresponding to disintegrin (DSIs) are absent in the retained fraction (Figure 2) and were not recognized in the ELISA-based immuno-profile (Figure 3A), which suggests a poor immunorecognition by INS antivenom over this protein family.

Additionally, the recognition pattern of INS antivenom over the crotapotins and the PLA_2_ from the crotoxin complex (subunits A and B, respectively) was highly variable and generally lower than the recognition over LAAOs and SVMPs (Figure 3).

### 2.2. Biological Activities of Venom and Neutralization Assays

The biological activity of *C. d. cumanensis* venom was evaluated. The minimum doses of biological activities were: 119.44 ± 11.15 μg for indirect hemolysis (MiHD); 5.89 ± 0.32 μg for coagulant activity (MCD) and 107.76 ± 4.84 μg for proteolytic activity (MPD). In the case of PLA_2_ activity on 4-NOBA, 50 μg represents the point before the enzyme reached its maximum activity. These quantities were used to test the neutralizing capacity of the antivenom.

Afterward, the neutralization assays showed that the antivenom did not have an effect over the indirect hemolytic and PLA_2_ activities, contrary to what was observed with the coagulant and proteolytic activities. Therefore, the statistical tests were carried out with the results of the neutralization assays over the coagulant and proteolytic activities. Firstly, the Shapiro–Wilk test showed that the results of the neutralization assays over the proteolytic activity did not follow a normal distribution (with the 100 μL dose a W = 0.7520 and a *p*-value = 0.0311 were obtained).

**Figure 4:**
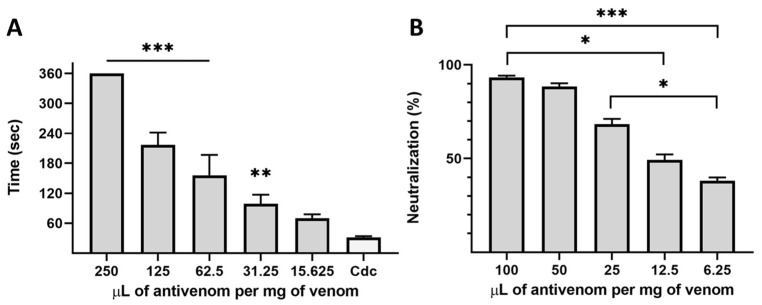
INS antivenom neutralization of biological activities. In (**A**), antivenom inhibition over *C. d. cumanensis* venom coagulant activity. In this case, three repetitions were done (*n* = 3) and the treatments were compared to a control that only contained venom (Cdc). A Tukey test for normally distributed data was applied, showing statistical differences between Cdc and 31.25 (*p* = 0.0088), and between Cdc and higher volumes of antivenom (*p* < 0.0001). In (**B**), antivenom neutralization capacity over the proteolytic activity of the venom is shown, in this case, all the treatments were compared between them (*n* = 5). A Tukey test was applied, indicating that the comparisons between 25–6.25 (*p* = 0.0127), 100–12.5 (*p* = 0.0127), and 100–6.25 (*p* = 0.0002) were significantly different. In each case, the statistically significant differences are shown as *, **, and ***, and represent a *p*-value smaller than 0.05, 0.01, and 0.001, respectively.

Then, a significant difference in the coagulation time was observed since the addition of 31.25 μL of antivenom per mg of venom, reaching its total inhibition at 250 μL/mg (Figure 4A). And, similarly, the neutralization capacity of the proteolytic activity was nearly 40–50% since the addition of the lowest antivenom quantities tested and 90% of the neutralization was achieved at 50–100 μL of antivenom per mg of venom (Figure 4B). The INS polyvalent antivenom was proved to neutralize the coagulant and proteolytic activities of *C. d. cumanensis* venom while, at the tested amounts, it was unable to inhibit the indirect hemolytic and PLA_2_ activities. However, the venom lethality was completely neutralized by INS antivenom, with an ED50 of 962 μL/mg venom. These results may be due to the amount of venom used in each test. For the inhibition of indirect hemolytic and PLA_2_ activities, we used 119.44 μg (one MiHD) and 50.0 μg, respectively, whereas, for the neutralization of lethal activity, we used 7.2 μg/mice (4 LD_50_). In the In vivo assay, antivenom was challenged with a low dose of venom. Thus, their neutralization capacity was higher.

## 3. Discussion

Antivenoms can recognize a wide variety of toxins, nevertheless, the venom proteins have different characteristics like molecular mass (MM), relative abundance, and conformation that determine the capacity of the immune system to produce antibodies against them, i.e., immunogenicity [36]. Therefore, antivenoms exert a differential immunorecognition ability over the venom proteins.

In this sense, the presence of poor immunogenic proteins in *Crotalus durissus* subspecies venoms such as crotamine, DSIs, and crotoxin -mainly its subunit A- and the high cross-reactivity of the venoms from *Bothrops* complex snake species [3,37,38,39] may explain why the *C. d. cumanensis* venom obtained the lowest level of immunorecognition (Figure 1). Even more, immunoreactivity assessments results showed a recognition ability pattern of the INS polyvalent antivenom lying towards the recognition of *C. d. cumanensis* venom proteins with high molecular mass (LAAO, SP, SVMP) whereas the low molecular mass proteins were poorly recognized (Figure 2 and Figure 3), as it has previously been observed with *Dendroaspis polylepis*, *Pseudonaja*, and various *Crotalus* species venoms [40,41,42].

The poor recognition by INS antivenom over the DSIs observed in the immunoreactivity assessments (Figure 2 and Figure 3) has been also obtained for other South American *Crotalus durissus* subspecies, such as *C. d. terrificus*, *C. d. cascavella*, *C. d. collilineatus*, *C. d. ruruima*, and *C. d. cumanensis* from Venezuela [3,37] and some *Echis* and Bitis snake species [43]. It is probably attributed to the low molecular mass and globular compact structure of DSIs, which makes them poorly immunogenic. Some DSIs affect platelet aggregation, nevertheless, their role in the pathophysiology of envenoming remains unclear [43].

The crotoxin is the most lethal protein in the *C. d. cumanensis* venom, it is a heterodimer composed of an acidic non-enzymatic subunit (Crotapotin or CA, ~9.6 kDa) and a basic enzymatically active PLA_2_ subunit (CB, ~13 kDa), the CA acts as a chaperone while blocking the substrate access to the active site of the CB, lowering its catalytic activity [44], but increasing its specificity for presynaptic membrane from the neuromuscular junction. After that it provokes inhibition of the release of acetylcholine, inducing a flaccid paralysis [13,45,46,47]. In addition, it has been demonstrated that crotoxin can induce a conspicuous systemic myotoxicity (rhabdomyolysis) and affect renal function, provoking acute renal failure, which is the main cause of death in the patients that suffer a crotalid snakebite in South America [12,48,49,50,51]. In this way, the crotoxin turns into the main responsible toxin for the majority of the neurotoxic, myotoxic, and nephrotoxic effects on the patients. Therefore, due to its toxic and lethal effects, crotoxin is a key objective in the crotalid antivenom design in Colombia.

Nevertheless, the recognition pattern of INS antivenom over the subunits A and B of crotoxin observed was not the best, because in some cases the binding to crotapotins and the PLA_2_ from the crotoxin complex was lower than the recognition over other non-lethal toxins in the *C. d. cumanensis* venom, such as LAAO, SVMPs and SPs (Figure 2 and Figure 3). This finding was also observed in the antivenomic studies of other *Crotalus durissus* subspecies, indicating the low immunogenic potential of crotoxin subunits and insufficient amount of antibodies against this toxin that is about 50% of the whole venom in *Crotalus durissus* venoms [3,13,37]. Nonetheless, another explanation for this result could be the saturation of the matrix-containing antibodies from antivenom which is a disadvantage of second-generation antivenomics that has been solved by the third-generation antivenomics [52]. Finally, the differences in CB recognition explain the inability of INS antivenom to neutralize PLA_2_ activity from *C. d. cumanensis* venom and its low ability to neutralize the lethality of this venom in comparison to what has been reported with other snake venoms [31,32,33].

To improve crotoxin recognition and neutralization, various techniques have been proved to be effective in the reduction of the crotoxin immunosuppressive activity while maintaining its immunogenicity, like heating [53], and using isolated CB subunit as the antigenic compound [54,55,56]. Nevertheless, novel promising approaches like the use of human-derived oligoclonal mixtures of antibodies [57], plant-derived toxin inhibitors [58,59], the immunization with recombinant consensus toxins [60,61], or DNA immunization [62] remain poorly explored.

The families of SP and SVMPs toxins were well recognized by INS antivenom (Figure 2 and Figure 3), which agrees with the good neutralization capacity of the INS antibodies over the biological activities exerted by these protein families (Figure 4). Similar findings were obtained in other antivenomic studies using commercial antivenoms against *Crotalus durissus* subspecies and other species [3,37,63,64]. Thus, it is demonstrated that the INS polyvalent antivenom efficiently neutralizes the coagulant and proteolytic activities which has been correlated to the venom hemorrhagic activity [65]. These SP- and SP/SVMP- based biological activities [65,66,67] are, probably, efficiently neutralized due to the high immunogenicity of these high MM toxins.

Previous studies that explored the composition of the *Crotalus* species venoms have shown two contrasting neurotoxic crotoxin- and hemorrhagic SVMP-predominant patterns that were correlated with changes in lethality (low and high median lethal doses, respectively) [68]. Although *C. d. cumanensis* -distributed in Venezuela and Colombia- has been shown to belong to the first pattern [13,14,69,70,71,72,73], some studies have shown strikingly divergent results -including the herein presented- in the in vitro biological activities [70,71,72,74].

The diverse venom phenotypes observed in the studied populations may be explained due to the geographical distribution of this snake, as it is considered a transition between the northern hemorrhagic and the southern neurotoxic venom patterns [3]. The high intra(sub)specific complexity and diversity here discussed implies that crotalid envenomation involving *C. d. cumanensis* may cause equally varied clinical manifestations that need to be neutralized by the antivenoms available along with its dispersal. Assessments of the neutralization capacity of antivenoms over the venoms provide a preclinical understanding of the inhibition of pathophysiological alterations of a snakebite envenoming [25].

A significant limitation of our study was the impossibility of performing a most advanced antivenomic technique. In this way, we can avoid the saturation of the matrix-containing antibodies from antivenom. In addition, third-generation antivenomics is more sensitive. Furthermore, another limitation was the lack of the isolated crotoxin complex to test the reactivity of INS antivenom against this neurotoxin.

## 4. Conclusions

The Colombian commercial INS polyvalent antivenom shows a restricted recognition ability over the crotoxin maybe to its immunosuppressive activity and low immunogenicity, thus encouraging the need for the exploration of new approaches in antivenom production. Also, a molecular mass-dependent pattern of venom toxins recognition showed that the low abundant SVMPs and SPs were more recognized than the highly abundant and poorly understood DSIs. INS polyvalent antivenom showed neutralizing capacity over the proteolytic and coagulant but not over the PLA_2_ activity of the *C. d. cumanensis* venom, which is in accordance with the molecular mass-dependent recognition pattern observed in the immunoreactivity assessment although other explanations should be further explored. Finally, similar studies should be carried out on other locally produced antivenoms to target the challenges in the improvement of antivenom therapy in Colombia.

## 5. Materials and Methods

### 5.1. Venoms and Antivenom

The venom of *C. d. cumanensis* was extracted from four adult individuals from the department of Meta (Colombia) maintained in the Serpentarium of Universidad de Antioquia in Medellín, Antioquia. The Colombian polyvalent anti-bothropic INS antivenom (batch 19SAPD1, expiry date June 2022) used in this study was manufactured by the Instituto Nacional de Salud and is composed of complete ammonium sulfate-precipitated horse IgG raised against *Bothrops* spp. and *C. d. cumanensis* venoms.

### 5.2. Immunoreactivity Assessments

#### 5.2.1. Antivenomics

The second-generation antivenomics approach [75], following a few variations [76], was carried out. For the preparation of immunoaffinity matrices, 2 mg of CNBr-activated SepharoseTM 4B in 3 mL of 1 mM HCl were packed into a column and washed with 10–15 matrix volumes of the same buffer, followed by two matrix volumes of coupling buffer (0.2 M NaHCO_3_, 0.5 M NaCl, pH 8.3) to adjust the pH to 7.0–8.0. INS polyvalent antivenom protein concentration was determined in a NanoDrop (Thermo Scientific (Waltham, MA, USA; with an absorbance at 280 nm). After this, 2 mL of antivenom in 6 mL of coupling buffer were incubated with the column matrix at 4 °C overnight using a spin wheel. The antivenom coupling yield was estimated using the absorbance values before and after the incubation. After the coupling, non-reacting groups were blocked with one matrix volume of blocking buffer (0.1 M Tris-HCl, pH 8.0) for 2 h at room temperature using a spin wheel. Affinity columns were washed 12 times alternating between two washing buffers with different pH (0.1 M sodium acetate, 0.5 M NaCl, pH 4.0; and 0.1 M Tris-HCl, 0.5 M NaCl, pH 8.0), using three volumes each time and finishing with the most basic.

After equilibration with five volumes of PBS (20 mM phosphate buffer, 135 mM NaCl, pH 7.4), the columns were incubated with 1 mg of *C. d. cumanenis* venom dissolved in ½ matrix volume of PBS and incubated at room temperature for 1–4 h using a spin wheel. The non-retained fractions of the columns were recovered with 2 matrix volumes of PBS, while the immunocaptured proteins were eluted with 3 column volumes of elution buffer (0.1 M glycine-HCl, pH 2.7) in a recipient with 900 μL of neutralization buffer (1 M Tris-HCl, pH 9.0). The retained and non-retained fractions were desalted and concentrated in 10,000 MWCO-Amicon^®^ centrifugal filters, then the concentrated fractions were analyzed by reverse-phase- HPLC using a C18 RESTEK column (250 mm × 4.6 mm, 5 µm particle size; RESTEK, Bellefonte, PA, USA) using a Shimadzu Priminence-20A chromatograph (Columbia, SC, USA) with protein detection at 215 mm. Elution was performed following previous specifications [77], with a 1 mL/min flow rate developed with a linear gradient of 0.1% trifluoroacetic acid (solution A) and acetonitrile (solution B) as follows: 0% B isocratically for 5 min; 0–15% B for 10 min; 15–45% B for 60 min; 45–70% B for 10 min; and 70% B for 5 min. In addition, the whole venom RP-HPLC profile was also obtained using 1 mg. Finally, to identify the proteins in the fractions in the resulting chromatograms the elution times were compared and associated with those obtained and identified by mass spectrometry in a previous study [13].

#### 5.2.2. Enzyme-Linked Immunosorbent Assay (ELISA)

For this assay, three different experimental designs were carried out: first, a *C. d. cumanensis* whole venom immunorecognition assessment with INS antivenom serial dilutions from 1:300 to 1:72,900; second, an assay with *C. d. cumanensis* venom fractions obtained from a whole venom RP-HPLC (with elution parameters as above) against a 1:900 antivenom dilution; and third, an immunorecognition assessment of a 1:900 INS antivenom dilution over venoms of *C. d. cumanensis*, *Bothrops asper*, *Bothrops atrox*, *Bothrops punctatus*, *Bothriechis schlegelii* and *Lachesis acrochorda*. In the first case, the experiment was performed in triplicates, while in the other two cases the experiments were performed in duplicates.

Firstly, 1 μg of substrate (whole venom or venom fraction) diluted in 100 μL of coating buffer (0.1 M Tris, 0.15 M NaCl, pH 9.0) was added in a well of a 96-well microplate and incubated for 16 h. Then, the content was discarded and 100 μL of blocking buffer (1% bovine albumin in PBS) was added and kept at room temperature for 60 min. The plates were washed five times with washing buffer (0.05% Tween-PBS, pH 7.2) and 100 μL of the correspondent antivenom dilution was incubated at room temperature for 2 h. The plates were washed five times and 100 μL of the 1:8000 anti-immunoglobulin/enzyme conjugate (diluted in 1% bovine albumin PBS) were added, and then the plates were incubated at room temperature for 2 h. After this, the microplates were washed five times one more time and 100 μL of peroxidase substrate (2 mg/mL OPD diluted in 0.1 M sodium citrate, pH 5.0; 4 μL of 30% H_2_O_2_ per 10 mL of final solution) were added. Finally, the absorbance was measured at 490 nm in a Multiskan sky spectrophotometer from Thermo Scientific (Waltham, MA, USA).

### 5.3. Biological Activities of Venom

#### 5.3.1. Coagulant Activity

The methodology proposed by a previous study [78] was followed. Various amounts of venom (10, 5, 2.5, 1.25, and 0.625 μg) dissolved in 50 μL of PBS were added to 200 μL of citrated frozen plasma obtained from the “Clínica León XIII” blood bank of The Universidad de Antioquia and previously incubated at 37 °C. The time that the plasma lasted to coagulate after the venom addition was recorded and the venom dose that induces coagulation in 60 s (the minimum coagulant dose, MCD) was estimated. For the positive and negative controls, 1 μg of *Bothrops asper* venom (equivalent to 1 MCD) and 50 μL PBS were tested, respectively. The experiments were performed in triplicate.

#### 5.3.2. Indirect Hemolysis

The model proposed by Habermann and Hardt [79] with modifications by Gutiérrez et al. [80] was applied. The minimum indirect hemolytic dose (MiHD) was defined as the venom dose that produced a 20 mm diameter hemolysis halo after 20 h of incubation. For this test, plates with a 0.8% agarose gel containing 250 μL of CaCl_2_ 0.01 M, 300 μL of egg yolk, and 300 μL of 100% erythrocytes were prepared. Then, different venom doses (120, 60, 30, 15, and 7.5 μg) in 16 μL of PBS were added in triplicate in wells equidistantly punched in the gel. For the control, 16 μL of PBS were tested. After the incubation, the hemolysis halo was measured and the MiHD estimated. The erythrocytes were obtained from the “Clínica León XIII” blood bank of The Universidad de Antioquia.

#### 5.3.3. PLA_2_ Activity

Phospholipase A_2_ activity on 4-nitro-3-octanoyloxy-benzoic acid (4-NOBA) chromogenic substrate was measured, as proposed by Cho and Kézdy [81] and Holzer and Mackessy [82], using the Ponce-Soto et al. [83] modification for 96-well plates. For the application of this test, different amounts of venom (100, 50, 25, 12.5, 6.25, and 3.125 μg) were diluted in 25 μL of NOBA buffer (10 mM Tris, 10 mM CaCl_2_, 100 mM NaCl, pH 8.0) and added to the wells along with 25 μL of 1 μg/μL 4-NOBA, and 200 μL of NOBA buffer. Then, the plate was incubated at 37 °C for 60 min and the absorbances were recorded at 405 nm in a Multiskan sky spectrophotometer from Thermo Scientific (Waltham, MA, USA). The PLA_2_ activity on the substrate was measured as the difference in the absorbance change between the control (NOBA buffer and substrate) and each treatment. The experiments were performed in triplicate.

#### 5.3.4. Proteolytic Activity

The proteolytic activity was determined according to Wang et al. [84] with some modifications. First, a 10 mg/mL solution of Azocasein (Sigma-Aldrich, St. Louis, MO, USA) in proteolysis buffer (25 mM Tris-HCl, 0.15 M NaCl, 5 mM CaCl_2_, pH 7.4) was prepared. Then, 20μL with various amounts of venom (200, 100, 50, 25, and 12.5 μg) were added to 100 μL of azocasein solution, which was subsequently incubated at 37 °C for 90 min. After this, the reaction was stopped by the addition of 200 μL of trichloroacetic acid, and the vials were centrifuged at 2000 rpm for 5 min. Supernatant aliquots (100 μL) were placed in ELISA plates and mixed with an equal volume of 0.5 M NaOH. Finally, the absorbance was measured at 450 nm in a Multiskan sky spectrophotometer from Thermo Scientific (Waltham, MA, USA). The minimum proteolytic dose (MPD) was estimated as the venom dose that induces a 0.2 change in absorbance in comparison with the control (proteolysis buffer without venom). The experiments were performed in triplicate.

#### 5.3.5. Neutralization of In-Vitro Assays

For determining the neutralization capacity of antivenoms over toxic activities of the venom, a constant dose of venom for each test was incubated (1 MCD, 1 MIHD, 1 MPD, and 50 μg for PLA_2_ activity) with variable amounts of antivenom (measured in μL of antivenom per mg of venom) at 37 °C for 30 min For the case of the neutralization of the proteolytic activity, a neutralization capacity percentage was calculated based on the proportion of the activity inhibited by each amount of antivenom compared with the activity of the venom alone. The experiments were performed in triplicate.

#### 5.3.6. Neutralization of Lethality

Animal experiments were performed in Swiss-Webster mice of both sexes and with 18–20 g of body weight and were carried out in accordance with the guidelines of the Ethics Committee of Universidad de Antioquia (License No.110 of 2017). A fixed-dose of *C. d cumanesis* venom corresponding to 4 LD_50_ (median lethal dose) was mixed and incubated with variable doses of INS antivenom and then injected by intraperitoneal route to groups of three mice. After 48 h the deaths were recorded, and the results were analyzed by a probits function. The neutralization was expressed as the median effective dose (ED_50_), which indicates the doses of antivenom required to neutralize one mg of venom. A control group injected with venom alone was used.

### 5.4. Statistical Analysis

Firstly, to determine if the data sets adjusted to a normal distribution, a Shapiro–Wilk test was applied (alpha was established at 0.05). Then, an ANOVA test followed by a Tukey test was applied (compared to a control or between all treatments, depending on the case). If the distribution was not normal, a non-parametric Kruskal-Wallis test followed by a Tukey test was applied.

## Data Availability

Not applicable.
